# “It’s better to have three brains working instead of one”: a qualitative study of building therapeutic alliance with family members of critically ill patients

**DOI:** 10.1186/s12913-018-3341-1

**Published:** 2018-07-09

**Authors:** Csilla Kalocsai, Andre Amaral, Dominique Piquette, Grace Walter, Shelly P. Dev, Paul Taylor, James Downar, Lesley Gotlib Conn

**Affiliations:** 1 Trauma, Emergency and Critical Care Research, Evaluative Clinical Sciences, Sunnybrook Research Institute, Toronto, Canada; 2 Interdepartmental Division of Critical Care, University of Toronto, Toronto, Canada; 3 Sunnybrook Health Sciences Centre, Toronto, Canada; 4 Palliative Medicine, University of Toronto, Toronto, Canada; 5 Patient/Client and Family Education, Centre for Mental Health and Addiction, 33 Russell Street, Toronto, Ontario M5S 3M1 Canada

**Keywords:** Therapeutic alliance, Communication, Collaboration, Decision-making, shared, Family, Intensive care units, Qualitative research

## Abstract

**Background:**

Studies in the intensive care unit (ICU) suggest that better communication between families of critically ill patients and healthcare providers is needed; however, most randomized trials targeting interventions to improve communication have failed to achieve family-centered outcomes. We aim to offer a novel analysis of the complexities involved in building positive family-provider relationships in the ICU through the consideration of not only communication but other important aspects of family-provider interactions, including family integration, collaboration, and empowerment. Our goal is to explore family members’ perspectives on the enablers and challenges to establishing therapeutic alliance with ICU physicians and nurses.

**Methods:**

We used the concept of therapeutic alliance as an organizational and analytic tool to conduct an interview-based qualitative study in a 20-bed adult medical-surgical ICU in an academic hospital in Toronto, Canada. Nineteen family members of critically ill patients who acted as substitute decision-makers and/or regularly interacted with ICU providers were interviewed. Participants were sampled purposefully to ensure maximum variation along predetermined criteria. A hybrid inductive-deductive approach to analysis was used.

**Results:**

Participating family members highlighted the complementary roles and practices of ICU nurses and physicians in building therapeutic alliance. They reported how both provider groups had profession specific and shared contributions to foster family communication, integration, and collaboration, while physicians played a key role in family empowerment. Families’ lack of familiarity with ICU personnel and processes, physicians’ sporadic availability and use of medical jargon during rounds, however, reinforced long established power differences between lay families and expert physicians and challenged family integration. Family members also identified informal interactions as missed opportunities for relationship-building with physicians. While informal interactions with nurses at the bedside facilitated therapeutic alliance, inconsistent and ad-hoc interactions related to routine decision-making hindered family empowerment.

**Conclusions:**

Multiple opportunities exist to improve family-provider relationships in the ICU.

The four dimensions of therapeutic alliance prove analytically useful to highlight those aspects that work well and need improvement, such as in the areas of family integration and empowerment.

**Electronic supplementary material:**

The online version of this article (10.1186/s12913-018-3341-1) contains supplementary material, which is available to authorized users.

## Background

Family members of critically ill patients value high-quality communication with the health care team [1]. Where family-provider communication has been suboptimal, patient-, family-, and staff-centered outcomes have suffered [1–8]. Patient safety and quality of care, family satisfaction and mental health, and staff wellness are affected [1–13]. Increasing healthcare costs are also associated with poor family-provider communication. These studies suggest that better communication between families and healthcare providers is needed in the intensive care unit (ICU) [3–8], yet most randomized trials targeting interventions to improve communication have failed or paradoxically led to worsening symptoms in family-centered outcomes [14–18]. This may be due to the limited focus of these interventions on communication regarding end-of-life care during formal, scheduled family meetings. While end-of-life care is an extremely important issue, family-provider interaction frequently begins well before end-of-life conversations are held or needed. In contrast, other fundamental dimensions of family-provider interactions, such as family daily involvement in care [10, 17–22], trust and support [1, 21–23], and family empowerment [1, 10, 19, 23, 24], receive relatively little scholarly attention.

By considering the multidimensionality and evolving nature of family-provider interactions, this study offers an examination of the complexities through which positive family-provider relationships may be established. We have drawn upon the concept of *therapeutic alliance*, which focuses on interrelated and essential dimensions of relationship-building between ICU families and providers: communication, integration, collaboration, and empowerment [25–27]. Therapeutic alliance is an ideal relationship that emerges between patients (or families as surrogates) and providers when conditions of these four distinct dimensions are met: a) effective information exchange and empathetic communication; b) integration of families into the care team through equalization of power by providers; c) collaboration between families and providers in establishing mutually agreed goals; and d) empowerment of families by providers to partner in decision-making [25–27]. While it is analytically useful to distinguish among these four dimensions for a holistic and complex inquiry into family-provider interactions and relationship-building, these dimensions are interrelated, as illustrated by their names and definitions. For example, integration and empowerment intersect partially, as integration is defined by the equalization of power between families and providers, and empowerment is the partnering in decision-making, that is, a particular type of integration. Therefore, integration can be established through empowerment, just as collaboration develops through communication.

Therapeutic alliance is associated with better outcomes [28–30]. Strong therapeutic alliance is related to emotional acceptance of terminal illness and less aggressive ICU care among patients with advanced cancer [29]. A recent study of family members of critically ill patients observed that therapeutic alliance was associated with families’ perception of increased patient-centered care [31]. However, the question of *how positive relationships are actually built*, or what behaviors, attitudes and practices contribute to therapeutic alliance in the ICU remains unexamined. In order to develop an in-depth understanding of the dynamic and evolving social relationships between families and providers during an ICU stay, we have taken a qualitative approach. This study explores family members’ perspectives on the enablers and challenges to positive relationship-building with ICU physicians and nurses with a view to further understand and optimally support patient- and family-centered outcomes.

## Methods

### Study design and setting

We conducted a qualitative study, using semi-structured interviews to explore families’ experiences and perspectives of how positive relationships are built with critical care providers. We followed a constructivist paradigm and focused our inquiry on the lived experience and the various ways in which families understand or make sense of their experiences of provider-family relationships [32].

The study took place in a 20-bed adult medical-surgical ICU of an academic hospital in Toronto. Sixteen staff physicians work on a weekly rotation schedule, with two covering physicians dividing patient care responsibilities in the unit of study. The two covering staff physicians also service other ICUs located in other parts of the hospital, and thus each cares for between 14 and 20 patients per physician at any given time. Clinical fellows (physicians certified in a base specialty who do further training in critical care) and residents (medical trainees enrolled in various specialty programs, including surgery, anaesthesia, emergency and general medicine) also participate in the care of ICU patients with variable assignment length to given patients (e.g.: for a day, a night, a week). One fellow and two residents are responsible for nighttime coverage. The ICU is staffed with nurses working 12-h shifts, with a 1:1 nurse to patient ratio. Other healthcare providers in the ICU include social workers, consultant physicians, pharmacists, dietitians, physiotherapists, respiratory therapists, speech-language pathologists and spiritual care providers. The study focuses on families’ interactions with physicians and nurses, because we only received limited data on other providers.

### Participants and sampling

We used a purposive sampling strategy with criterion and maximum variation sampling techniques [33]. English-speaking family members of ICU patients undergoing prolonged ventilation with a minimum stay of 48 h and a maximum stay of 1 month were eligible. We recruited family members of a diverse patient population and belonging to a wide range of demographics. We interviewed families who were present during daytime hours as well as those who were not available during daytime or did not visit regularly.

### Data collection and analysis

Interviews were conducted in-person in the family meeting room of the ICU. The interview guide was based on research team expertise and a literature review on therapeutic alliance, communication and conflict in the ICU (Additional file 1). A cultural anthropologist with prior experience in health services research (CK) interviewed all participants. The interviews lasted for 30 to 80 min, were audio-recorded and transcribed verbatim. Originally, we planned to include only substitute decision makers (SDM), who are legally required to make decisions and to give consent on behalf of their incapacitated relatives, but found that in some instances other family members interacted with providers more frequently, and thus those relatives were also included in the study.

The analysis was iterative and followed a hybrid inductive-deductive approach [34]. Three investigators (CK, LGC, DP) read and coded the first six transcripts independently to determine major themes and identify areas for additional inquiry. Through discussions with the research team, the primary author (CK) analyzed the remaining transcripts to refine and expand the coding scheme using the constant comparison method. Data collection ceased when saturation was reached [33]. Following an initial inductive coding strategy and to guide further interpretation, we used the four dimensions of the therapeutic alliance concept as an organizational and analytic tool. NVivo 10 was used for data storage and management.

## Results

We interviewed 19 family members, 17 SDMs and 2 non-SDMs between October 2014 and February 2015 (Tables 1 and 2). Nurses’ and physicians’ complementary roles and practices fostered therapeutic alliance across 3 dimensions: communication, integration, and collaboration. Family members perceived that only physicians were positioned to empower them to participate in decision-making. Study participants also revealed barriers and missed opportunities to establishing therapeutic alliance, especially in the integration and empowerment dimensions. Therapeutic alliance was challenged by certain physician practices that unintentionally perpetuated power differences with families, alienating them and hindering family integration. Missed opportunities for informal interactions between physicians and family members further challenged family integration and empowerment and thus the establishment of positive family-provider relationships. These findings are elaborated below.

**Table 1:** Characteristics of family participants (*n* = 19)

Characteristics	Value
Female, n (%)	14 (74%)
Age, mean (range)	47 (20–72)
Race/Ethnicity	
White, n (%)	13 (68%)
Black, n (%)	2 (10%)
Other, n (%)	4 (21%)
Religion	
Christian, n (%)	13 (68%)
Other, n (%)	4 (21%)
Non-religious, n	2 (10%)
Substitute Decision Maker, n (%)	17 (89%)
Relationship to Patient	
Spouse, n (%)	5 (26%)
Child, n (%)	9 (47%)
Parent, n (%)	4 (21%)
Other, n (%)	1 (5%)
Time of visitation	
Daytime visitation, n (%)	16 (84%)
After hours and non-regular visitation, n (%)	3 (16%)

**Table 2:** Characteristics of patients (*n* = 19)

Characteristics	Value
Female, n (%)	9 (47%)
Age, mean (range)	54 (17–87)
Reasons for admission, n (%)	
Neuro and TBI	8 (42%)
Respiratory	4 (21%)
Sepsis	2 (10-%)
Postoperative elective	1 (5%)
Trauma	4 (21%)
Length of Stay at Time of Interview	
2 to 4 days	2 (10%)
5 to 10 days	8 (42%)
More than 11 days	9 (47%)

### Communication: Exchanging information and providing empathy

Through distinct and shared communication practices nurses and physicians built positive relationships with family members. Physicians provided medical information, while nurses facilitated the understanding of medical updates and provided sustained emotional support with frequent, reliable, clear, and empathetic communication throughout the day.

Family members recognized both nurses and physicians as valuable sources of information regarding the patient’s condition and care:

*“Many people like to be informed, they like to know what’s going on so they don’t feel lost and helpless*.”

Beyond content, communication style and delivery mattered tremendously to families and, when done with clarity and empathy, communication positively contributed to building relationships. Participants appreciated “*honest*” and sincere information during bedside updates or family meetings, and sometimes used the term “*no sugar-coating*” to describe effective communication with both nurses and physicians.

Proactively sharing and frequently repeating information helped families. “*Forthcoming*” providers were valued by families who were concerned with asking too many questions, and by those who struggled to ask any questions: “*How do you know what questions to ask the doctor?*” Families felt reassured by repetitive information provided by nurses and physicians, highlighting the importance of their complementary practices:

“*I find the doctor corroborates what the nurse said*. *It is nice to hear it again*.”

Family members perceived nurses to be especially good at explaining clinical information in simple, jargon-free language. As they put it, nurses talked “*in our language*.” Nurses were perceived as a reliable source of answers to questions emerging among family members during informal updates, and bedside rounds.

Families recognized physicians’ compassionate communication during family meetings focused on end-of-life issues, but viewed nurses as expressing empathy more consistently throughout the patients’ ICU stay. They not only appreciated how nurses respectfully treated unconscious patients, but how they addressed family distress: “*They can even comfort me and help me get through this*.” Nurses built emotional connections with family members through concrete gestures, such as asking how they were doing, bringing an extra chair, or offering a bottle of water. As one family member put it:

*“I don’t know if anyone realizes just how important that personal touch is. Nurses are there to do the job and they do it well, but they also give you a hug and a smile. And reassurance - they aren’t miracle workers but you’d almost think that they were.”*

### Integration: Equalizing power differences

Although some positive aspects of communication were recognized by our participants in both nurses and doctors, others were seen as specific to one professional group. Physicians were uniquely recognized for integrating families into the care team by encouraging them to attend daily interdisciplinary rounds and to participate in the exchange of information and decision-making regarding patient care. Rounds were recognized as the most reliable opportunity to speak with physicians, and many family members scheduled their ICU visits in order to attend this morning activity. Some of these families reported that staff’s questions made them feel valued: “*They know that I know my mom best*.” Others appreciated receiving a brief update about their loved one at the end of the team’s discussion. Some families found that attending rounds helped to *“build trust and community*.”

However, family members who gained access to interdisciplinary rounds did not necessarily feel integral to patient care. Integration was challenging for some families because of the technical discussion occurring during rounds. Many families reported that “*most of what I hear I don’t understand*.” Inaccessible language typically used by interdisciplinary team members made rounds “*overwhelming*” and “*intimidating*” for family members.

While physicians were mostly available during daily rounds and formal family meetings, family members found nurses to be more continuously available at the bedside. They appreciated having the same nurse for a few consecutive days: “*It’s nice to have the face that I know and can talk to easily*.” They often knew the nurses by name, and with prolonged patient ICU stay, families “*really got to know*” them. Families acknowledged that nurses’ availability was limited due to scheduling, but valued how nurses integrated them by recognition and reconnection, as they came back to the bedside to give them a hug, or chatted with them while taking care of a patient nearby. Nurses facilitated family members’ integration by acting as an intermediary between the family and other members of the health care team. They sought out answers to family questions, and involved families in direct patient care delivery, for example, putting lotion on their loved one’s hands, wetting their lips, and accompanying their loved one on the way to the operating room.

By contrast, physicians’ sporadic presence at the bedside or in the unit was perceived to hinder family integration into the team. Many families found, for example, “*It’s a hit and miss if you see a doctor*.” Some families complained that staff physicians did not always come back to update them after rounds as promised. Those who visited exclusively during evenings or weekends reported never communicating with the physician in charge:

*“We found out who was responsible, but never had the opportunity to meet him in person. It was always residents.”*

Scheduling practices and the teaching orientation of the clinical environment were some of the structural factors that shaped and constrained physicians’ work: staff physicians rotated weekly, handovers occurred twice daily between daytime and nighttime clinical teams, and multiple providers were involved in the daily care of each patient. This large and often changing team of physicians confused families:

*“I feel like so many different people are with her all the time. It’s not like one main person. So I get confused half of the time.”*

A family member thus compared her experience with the care team to “*a revolving door*.” Many participants admitted to not knowing who the physician in charge was, or identified the wrong individual:

“*I still don’t know which doctor is in charge of him. I just know [Dr. XY] did the surgery. But I think it’s his students who are actually doing the rounds but I don’t know if there is actually one student in charge of him*.”

It was also common for family members to mistake medical trainees for nurses. A family member summed up the problem of staff identification as follows: “*I wouldn’t know who is a doctor, everyone who has a stethoscope?*” This structure added to families’ confusion, and was enhanced by doctors frequently failing to introduce themselves to the family at the bedside: “*It would be nice just to say hi, I’m so-and-so*.”

Yet, families assumed their share of responsibility for the shortcomings in physician-family interactions by recognizing their own passivity in requesting updates and pointing to their own limited availability. They also believed that physicians’ limited presence was at times justifiable because of their busy schedules and competing priorities: “*They would rather spend the time taking care of the patients than telling me what’s going on.*”

Access to providers and to the patient was therefore an essential, and sometimes limiting, factor in family integration in patient care, but was not in itself sufficient to family integration. The understanding of ICU roles and rules, norms and practices, language and structures, however, proved essential to full family integration but often remained incomplete.

### Collaboration: Establishing mutually agreed goals

Family members reported that advocating and proactively working with nurses and physicians enhanced their feeling of collaboration with the ICU team: “*It’s better to have three brains working instead of one*.” Families positively reflected on formal, scheduled family meetings with physicians aimed at reassessing goals of care. They highlighted how doctors “*made the time to speak*” with them, discussed patient values and hopes, and brought the bad news “*apologetically*.” Physicians were perceived as helpful for answering questions and advising on decision-making related to life-supporting therapies. In doing so, physicians often expressed uncertainty and empathy.

As families advocated for their loved ones, they valued how nurses listened to their simple suggestions, such as which side their loved one liked to lie on. They also appreciated sharing personal knowledge of the patient, for example, the existence of allergies, need for pain medication, or discomfort caused by procedures. Families liked when nurses seriously considered what they suggested: *“So they would really not dismiss any of my comments, they would say, okay let’s see together and get things sorted out.”*

Addressing family members’ knowledge of the patient was found imperative to establish daily and bigger picture goals. While physicians collaborated with families to establish complex, overarching care goals, nurses collaborated with family members to reach everyday goals.

### Empowerment: Partnering in decision-making

Physicians played a key role in empowering families in sharing decision-making during formal and scheduled family meetings about a wide range of medical decisions, from tracheostomy to blood transfusion and surgery. Family members described how clear and understandable explanations about the care plan helped them make decisions: “*He laid it out very well so that we could make more of an informed decision now.”* Families also appreciated when physicians respected their decisions even if they disagreed with the plan: “*They did not write up the order, which I think is really positive because they could tell by my reaction that I wasn’t comfortable with it*.”

Formal, scheduled meetings and end-of-life discussions were therefore described positively, but informal bedside or phone interactions with physicians appeared less satisfactory for families, representing a missed opportunity for family empowerment. A number of families described inconsistencies in how physicians sought their input to make smaller-scale decisions related to the daily care of patient. One participant complained that a physician called her to consent for a blood transfusion, though on previous occasions the patient received the same treatment without her consent. Another participant perceived that clinicians preferentially engaged them when physically present at the bedside, “*because they were already in the room and wanted to do the procedure.*” Similarly, another participant found it upsetting to be called while traveling by bus to the hospital in order to make a hasty decision about a non-urgent procedure that later proved unnecessary:

*“They thought he was bleeding in his stomach so they wanted to put a tube down his throat to see about that. It was kind of bad timing. I was on the bus and I really couldn’t hear. The conversation probably went well but I was caught off-guard. …I should have said, I’m on my way, I’ll be there in a minute and can we hold off 5 min because I would like to ask a question first before you do this?”*

These inconsistent ways of seeking family input for decision-making during informal family-physician interactions left families questioning how truly valued their contribution to decision-making was, and thus challenged family empowerment. They created confusion about their role as SDMs, leading them to decisional regret, loss of trust, and the feeling of exclusion from the care team.

## Discussion

Family members experience many facilitators and barriers to building therapeutic alliance in the ICU. We found this to be a complex and dynamic process, evolving through a series of formal and informal social interactions between families and different healthcare providers. We observed three findings: First, nurses and physicians occupy complementary roles and perform complementary practices through which they together foster positive relationships with family members. Family members described their complementary practices in communication, integration, and collaboration, and highlighted physicians’ role in empowering them in decision-making. Second, families’ lack of familiarity with ICU personnel and processes, physicians’ limited availability and use of medical jargon during rounds often reinforced long established power differences between lay families and expert physicians, and thus challenged family integration and the establishment of therapeutic alliance. Third, families highlighted informal interactions as missed opportunities for physicians to build therapeutic alliance. While informal conversations and interactions with nurses at the bedside facilitated positive relationships, inconsistent and ad-hoc communication about decision-making led to a sense of family confusion, which challenged family empowerment and alienated them from the care team.

Our analysis shows that therapeutic alliance in the ICU is an interprofessional effort that relies on doctors and nurses in different ways. While the importance of interprofessional communication in this dyad is known, these findings uniquely highlight how families perceive and experience the profession specific *and* interprofessional strategies to communication, integration, collaboration, and empowerment. For example, repetition, corroboration and clarification are interprofessional communication strategies reflecting physicians’ and nurses’ collaboration in establishing therapeutic alliance with families. As a result, family members are reassured, supported, and kept informed. Previous research, however, has identified the need for an interprofessional strategy to improve family communication and decision-making [15, 35–38], with an emphasis on third-party facilitation [14, 15, 17, 18]. Our findings suggest that nurses and physicians are well positioned to share responsibility for fostering positive relationships with families, which could benefit both families and providers. Identifying the specific contributions that each profession brings to this relationship and effectively leveraging them appears as an important strategy to improve family-centered outcomes. Acknowledging one another’s contributions towards this shared goal could also provide a mechanism through which some of the interdisciplinary tensions between nurses and physicians, due to differences in professional cultures, medical dominance, and competing interests, can be mitigated in the ICU [39–43].

Nurses’ and physicians’ interactions with families, and their complementary roles and practices in building therapeutic alliance with them are heavily determined by structural factors, such as provider coverage models and the teaching orientation in the particular ICU. Such factors shape physician-family interaction as more sporadic, which can still achieve important benefits in the moment along the dimensions of communication, integration, collaboration and empowerment. In contrast, nurse-family interaction can become more longitudinal and consistent, conducive for empathetic communication and the establishment of an emotional bond. Although the inclusion of nurses in decision-making about goals of care is recommended by current guidelines, this recommendation is based on studies that employed a non-bedside, usually specially trained nurse [1]. Our findings, however, suggest that further research should explore and tap into the potential of bedside nurses in supporting families and achieving therapeutic alliance.

Findings related to integration offer insights into power relations operating between ICU providers and families. Inequalities have been previously explored across various clinical contexts [44, 45], but our findings bring attention to the complexity of power relations with families in the ICU. Daily rounds provide opportunities for family participation and integration into the team, and may reduce the historically established power differences between expert doctors and lay family. Some families, however, experienced discomfort and a sense of exclusion due to physicians’ use of technical language, revealing how family attendance on rounds does not necessarily lead to family-centered rounds [46, 47]. These bedside rounds were intended primarily to make decisions about patient care and teach medical trainees, but recently, along with the movement towards person-centered care, they were opened up for the inclusion of families. By observing how power differences are perpetuated through language [48], our study demonstrates and explains the challenge of family inclusion in daily rounds and the potential for unintended consequences. It thus expands on other studies that point to the increase in family anxiety during rounds without unpacking its source [1]. To achieve family integration through rounds, we echo the Society of Critical Care Medicine’s (SCCM) call for further research on the best ways to incorporate families into rounds beyond their mere presence [1].

Family members’ tendency to rationalize physicians’ limited presence by their busy schedules and competing priorities also reinforces hierarchical relations in family-provider interactions. Notwithstanding physicians’ intensive workflow, it is important to highlight how families’ justifications quickly subscribe to the culturally dominant narrative of physicians’ being too busy to spend time talking with them. Recognizing time as a differential resource, families lower their expectations about possible physician-family encounters and take up a more passive role. They unintentionally eclipse communication as physicians’ professional responsibility and curtail opportunities for their integration into the team. Such assumptions and associated behaviors point to the dynamics of physician-family interaction and how family members are complicit in the reproduction of power relations, posing a barrier to establishing positive relationships. The concept of therapeutic alliance overlaps with other important contemporary goals of health care, such as family-centered care and shared decision-making, all of which value egalitarian relations [45], but our analysis finds that they often remain ideals in a cultural context mediated by power.

Therapeutic alliance also provides a useful lens to understand the importance of informal encounters in shaping relationships in the ICU [20, 22, 49]. Families highlighted the value of nurses’ informal and consistent updates and other interactions, through which nurses effectively communicated and collaborated with families, and integrated them into the care team. In contrast, physicians’ sporadic presence and episodic communication patterns at formal, scheduled meetings constrained the relationships they built with families. Furthermore, inconsistencies in physicians’ informal interactions around consenting procedures often challenged family empowerment, impeding therapeutic alliance and possibly contributing to conflict. Although studies and guidelines to date have mostly focused on formal interactions, such as family meetings about end-of-life and other big decisions [1, 16, 17, 23, 50, 51], our findings call for the recognition that informal, ad hoc interactions with a range of providers over time are important part for establishing therapeutic alliance. Further research on how informal interactions influence the family-provider relationship is needed.

This study is novel in its qualitative approach to therapeutic alliance. It has used a broad sampling strategy to include family members of a diverse patient population in order to draw out commonalities in their experiences during the patient’s ICU stay. Further research is needed to understand how ICU providers build relationship with specific family groups, in particular those who are not English-speaking or those whose loved one belongs to medical versus surgical populations. The study is also unique in its focus on families’ interactions with nurses and physicians simultaneously, at the exclusion of participants’ experiences with other health care team members. Future work should explore family-provider interactions from both the families’ and all health care providers’ perspectives. While interviews were effective and insightful, direct observations of how interactions occur in specific contexts would provide additional richness to the analysis. Finally, findings represent the experiences of English-speaking families within a single academic ICU in Canada and may not be transferable to non-English speaking families and/or other ICUs.

## Conclusions

The four dimensions of therapeutic alliance prove analytically useful to highlight current strength in ICU provider-family relationships. We identified several opportunities to improve these relationships in the areas of family integration and empowerment. In our center, we have implemented a comprehensive informational website for families, an initiative that aligns with the SCCM’s family-centered care guideline for family education [1]. Our website aims to increase access to and understanding of ICU personnel and processes, and support families’ integration into care. We have sought to expand on the guideline’s recommendations on family-centered communication training for ICU clinicians by implementing role-playing exercises for critical care trainees to highlight the importance of informal family interactions that target family empowerment. Future efforts to build therapeutic alliance should also consider educational activities that enable physicians and other providers to reflect on the norms and practices that produce inequality across the provider-family dyad [52]. Further research is necessary to understand the best ways to integrate families into rounds, how to build on the complementary roles and practices of physicians and nurses, and how informal interactions shape and constrain family-provider relationships. Multiple interventions are likely needed to continually foster positive relationships, mitigate identified barriers and transform the ICU into a more interprofessional and family-centered therapeutic environment to enhance patient- and family outcomes.

## Additional file

Additional file 1 Interview Guide. This file contains the semi-structured interview guide that was used to guide discussions with research participants. (DOCX 17 kb)

## Abbreviation

ICU: Intensive care unit
