# Supplementary material for: “It’s better to have three brains working instead of one”: a qualitative study of building therapeutic alliance with family members of critically ill patients
Source: BMC Health Serv Res. 2018 Jul 9;18:533. doi: 10.1186/s12913-018-3341-1 (PMC6038351; doi:10.1186/s12913-018-3341-1)
Supplement: Supplementary file 1 — Interview Guide. This file contains the semi-structured interview guide that was used to guide discussions with research participants. (DOCX 17 kb) [file 12913_2018_3341_MOESM1_ESM.docx]

**Additional file 1**

**Title: “It’s Better to Have Three Brains Working Instead of One”: Building Therapeutic Alliance with Family Members of Critically Ill Patients**

**Interview Guide**

Thank you for your participation in the study. As you know, we are conducting research on interactions between family members and the health care team at the ICU. Through your description, I would like to understand your experiences of communicating and interacting with ICU providers since your loved one has been admitted here. I would like you to describe your thoughts and feelings in as much detail as possible.

Can you talk about your loved one’s admission to ICU?

Can you recall how you first met your loved one’s health care team?

How do you find the updates about your loved one’s condition and care, and talk about how each provider shares information?

Can you discuss how you make sense of what you are told and what have you found helpful?

How do you find bedside rounds?

Could you recall whether you have had discussions about treatment/test decisions and if so, how did you find them?

Could you tell me about your experience of having a family meeting that is usually scheduled to take place in the family room?

How has complications or uncertainties been communicated to you?

How has prognosis been discussed?

How has your loved one discharge/transfer been discussed with you?

If end-of-life care has been discussed, could you talk about your experience?

How has your role and involvement in the care been discussed?

What have you found especially helpful and challenging during your loved one’s stay in the ICU?

Do you have some suggestions for improving your communication and interaction with the health care team?

How would you feel about getting some general information about what happens in the ICU through a website? Why?
